# Computing the Volume, Surface Area, Mean, and Gaussian Curvatures of Molecules and Their Derivatives

**DOI:** 10.1021/acs.jcim.2c01346

**Published:** 2023-01-13

**Authors:** Patrice Koehl, Arseniy Akopyan, Herbert Edelsbrunner

**Affiliations:** † Department of Computer Science, University of California, Davis, California95616, United States; ‡ FORA Capital, Miami, Florida33131, United States; § IST Austria, 3400Klosterneuburg, Austria

## Abstract

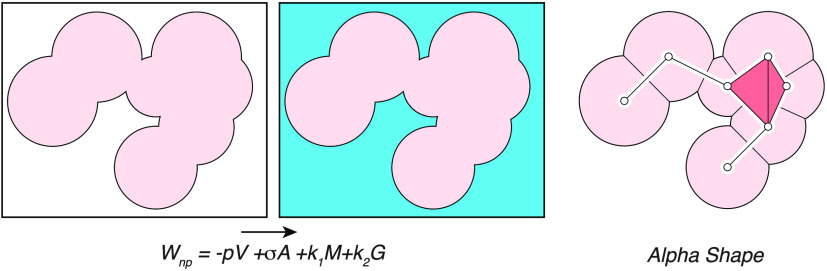

Geometry is crucial in our efforts to comprehend the structures and dynamics of biomolecules. For example, volume, surface area, and integrated mean and Gaussian curvature of the union of balls representing a molecule are used to quantify its interactions with the water surrounding it in the morphometric implicit solvent models. The Alpha Shape theory provides an accurate and reliable method for computing these geometric measures. In this paper, we derive homogeneous formulas for the expressions of these measures and their derivatives with respect to the atomic coordinates, and we provide algorithms that implement them into a new software package, AlphaMol. The only variables in these formulas are the interatomic distances, making them insensitive to translations and rotations. AlphaMol includes a sequential algorithm and a parallel algorithm. In the parallel version, we partition the atoms of the molecule of interest into 3D rectangular blocks, using a *kd*-tree algorithm. We then apply the sequential algorithm of AlphaMol to each block, augmented by a buffer zone to account for atoms whose ball representations may partially cover the block. The current parallel version of AlphaMol leads to a 20-fold speed-up compared to an independent serial implementation when using 32 processors. For instance, it takes 31 s to compute the geometric measures and derivatives of each atom in a viral capsid with more than 26 million atoms on 32 Intel processors running at 2.7 GHz. The presence of the buffer zones, however, leads to redundant computations, which ultimately limit the impact of using multiple processors. AlphaMol is available as an OpenSource software.

## Introduction

Biological nanomachines, such as proteins and nucleic acids, are essential for all cellular functions due to their abilities to store information, to provide transport to and out of the cell, to catalyze chemical reactions, and to interact and recognize ligands, among other things. Their functions are believed to be intimately related to their shapes (referred to as *structures*), as well as to the dynamics of these shapes. Our current knowledge of the structures and dynamics of large biomolecules remains inadequate. This is because only a few experimental techniques have the ability to gather structural data that are resolved in time and those that can are typically constrained to small length scales and to short time windows. Recently, new algorithms have been proposed for predicting the structures of proteins that have reached significant success. However, predicting and analyzing the dynamics of such structures are tasks that are still limited in scope, both with respect to time scales (usually microseconds to milli-seconds) and length scales (several nanometers for systems of up to hundred thousand atoms).

With the success of AlphaFold^1^ and its successor AlphaFold2,^2^ artificial intelligence has stormed into structural molecular biology in the recent years.^3,4^ This is software designed by the company DeepMind to predict the structure of a protein based on its sequence only. AlphaFold has refined numerous deep learning techniques to predict these structures at near experimental-scale resolution, inspiring experimental structural biologists to rethink the way they study the function and evolution of proteins, as well as their impact on diseases.^5−7^ AlphaFold’s achievement has been made possible by the wealth of information present in the Protein Data Bank,^8^ the database of experimentally determined protein structures (approximately 200,000 as of October 2022). In return, AlphaFold allowed for the prediction of millions of previously unknown protein structures, all available in an open database.^9^ There are, however, limitations to AlphaFold,^7^ which only generates single-ranked conformations for a protein. As such, it is currently unable to provide information on ensembles of conformations for a protein, which may arise if this protein is intrinsically disordered. AlphaFold does not solve the protein folding problem as it is inherently static. It does not capture conformational mechanisms such as allostery. The study of these mechanisms still relies on simulations of molecular dynamics.

The standard approach to simulating the dynamics of a biomolecule is to solve numerically the Newton equations associated with all its atoms. The step size in time required for finding accurate solutions to those differential equations is extremely small (in the order of a femtosecond), leading to the need to compute the energy of the molecular system under study a large number of times. One evaluation of the energy is of order *O*(*N* log  *N*), with *N* being the total number of atoms in the system (including the water molecules in the environment of the biomolecule). For large values of *N*, say in the millions, such a calculation and more importantly its repeats become computationally prohibitive. While it is possible to design hardware that is specific to such calculations and while many efforts are underway to improve the software that implement them^10−16^ (currently allowing for molecular dynamics simulations of systems with up to 100 million atoms^17−21^), parallel efforts are put into developing simplified models in which the number of atoms is reduced to make the calculation more tractable. Of particular interest is to replace the explicit solvent with a potential of mean force that mimics its effect on the molecule. This is akin to deriving and computing a solvation free energy, *W*_*sol*_, for the biomolecule. How to compute the nonpolar component of this solvation free energy is the topic of this paper. It is noteworthy that current versions of AlphaFold focus on the conformation of the protein alone, independent of its environment. Inclusion of solvation free energy to further refine the prediction is likely to be an addition in newer versions of the software, reinforcing the need to derive accurate and robust methods for computing such solvation free energies.

### A Morphometric Approach to the Nonpolar Solvation Free Energy

**Figure 1:**
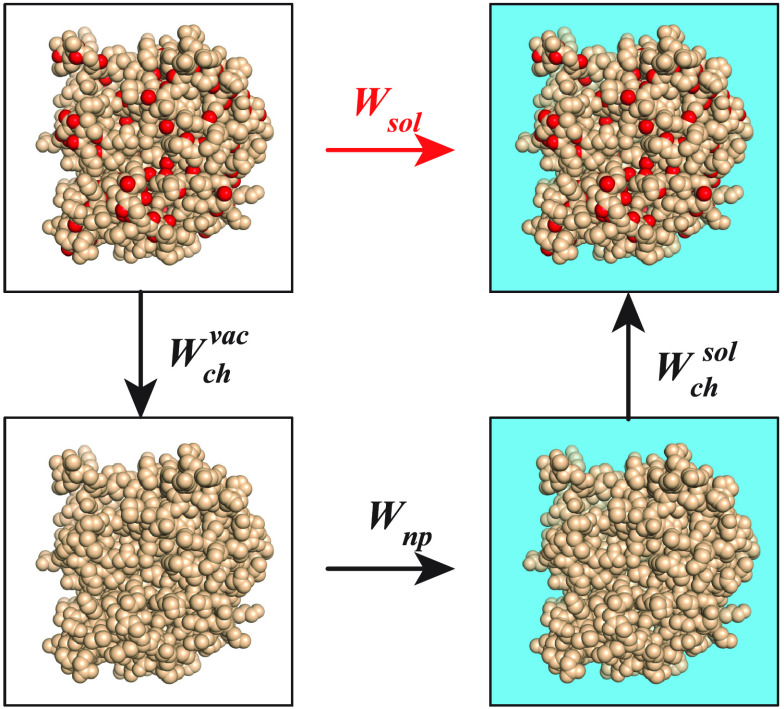
Computing the solvation free energy of a biomolecule. The solvation free energy, *W*_*sol*_, is defined as a mean force potential that quantifies the energy that is required to solvate a molecule. It consists of two parts: (i) a polar contribution, *W*_*pol*_, which accounts for the effects of the solvent on the charges of the biomolecule and (ii) a nonpolar contribution, *W*_*np*_, which accounts for the formation of a hole within the solvent so that it can fit the biomolecule as well as for the vdW interactions between the biomolecule and the solvent (at the surface of the biomolecule). These two parts are best described with a thermodynamics cycle. First, the charges on the biomolecule (symbolized by the red balls) are neutralized in vacuo. The corresponding free energy cost is referred to as *W*_*ch*_^*vac*^. Second, the corresponding neutral molecule is solvated, with a cost *W*_*np*_. Finally, the charges are added back to the molecule, now in solution, with an energetic cost *W*_*ch*_^*sol*^. The solvation free energy is the sum of those three contributions, namely,  (red arrow).

The solvation free energy *W*_*sol*_(*X*) of a biomolecule with conformation *X* is set to capture the presence of a cavity within the solvent that enables it to accommodate the biomolecule and the vdW interactions between the water molecules and the atoms at the surface of the biomolecule, as well as the interactions between the charged atoms of the biomolecule in the presence of water. The first two contributions define the nonpolar effect, *W*_*np*_, while the third one captures the polar effect, *W*_*pol*_. These effects are additive, namely, *W*_*sol*_ = *W*_*np*_ + *W*_*pol*_. They can be computed individually with the help of a thermodynamic cycle, as illustrated in Figure 1.

Eisenberg and McLachlan^22^ proposed an atomic break down of the computation of the nonpolar part of the solvation free energy of a biomolecule. In their model, each atom is represented with its accessible surface area, *ASA*,^23^ which is then scaled with a surface tensor factor referred to as atomic solvation parameter, or *ASP*, such that
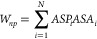
1The *ASP* is a signed number, positive for nonpolar atoms (large accessible surface areas are then penalized for such nonpolar atoms) and negative for polar atoms (i.e., favoring large accessible surface areas for them). This surface-only model, referred to as SA, is supported indirectly with the observation that the Gibbs free energy for transferring small compounds from nonaqueous liquids to water is linearly related to their accessible surface area. SA has become the preferred approach for studying the dynamics of a biomolecule with an implicit solvent, in conjunction with Poisson–Boltzmann (PBSA) or generalized Born (GBSA) .^24^ It is interesting, however, to think back on the fact that *W*_*np*_ accounts for two effects, namely, the hole formation in the solvent and the vdW interactions between the atoms of the biomolecule and the solvent molecules. While the latter occurs near the boundary between the biomolecule and the solvent and is therefore proportional to the accessible surface area of the molecule, the former is proportional to the volume of that molecule. This apparent contradiction between a surface area model only and the fact that *W*_*np*_ includes a volume-based contribution currently fuels a debate on the geometric nature of *W*_*np*_. Lum, Chandler, and Weeks for example have shown that *W*_*np*_ scales with the volume of the solute for small solutes, is proportional to the surface area for very large solutes, and should consider both geometric measures in between.^25^ This idea that *W*_*np*_ for a molecular system depends on surface area and volume is derived from scaled particle theory.^26−28^ It was shown to be a better representation of solvent effects than a surface-based solvation free energy.^29,30^ However, even a combined surface area and volume representation for *W*_*np*_ seems to be deficient to represent length-scale dependence of this energy.^31^ More recently, *W*_*np*_ has been expressed as a linear combination of the four morphometric measures of the molecule:^32^
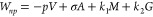
2In this equation, *V*, *A*, *M*, and *G* are the volume, surface area, mean curvature, and Gaussian curvature of the molecular system, while *p*, σ, and *k*_1_ and *k*_2_ are the pressure, surface tension, and bending rigidity parameters. This is under the assumption that the solvation free energy satisfies (i) *motion invariance*, namely, independence with respect to the location and orientation of the molecular system in space, (ii) *continuity*, basically that thermodynamics can be expressed in terms of geometry (a condition that is only violated for system whose size is similar to the size of the solvent), and (iii) *additivity*, i.e., that the energy of the union of two domains is the sum of the energy of the single domains subtracted by the energy of the intersection. This model has proved useful to study the solvation of proteins and their ligands.^33−35^

All the solvation models presented above consistently associate the nonpolar contribution to the solvation free energy of a molecule to the geometry of this molecule, the *continuity assumption*. In what follows, we discuss how different measures of this geometry, more specifically volume, surface area, mean curvature, and Gaussian curvature, and their positional derivatives can be computed efficiently, even for very large molecular systems.

### Computing Geometric Measures of Biomolecules

A union of balls, with each ball representing an atom, is a typical geometric representation of a molecule. Lee and Richards^36^ developed the first approach to computing the accessible surface area of a protein represented by such a union by first cutting it with a set of parallel planes. Shrake and Rupley^37^ proposed instead a Monte Carlo numerical integration method to compute regions of the surfaces of atoms that are accessible. Many efficient implementations of this method have been proposed, including the use of look-up tables,^38^ as well as of algorithms that make use of the parallel architecture of computer central processing units (CPU) .^39,40^ All those methods have been expanded to compute also the volume of a union of balls.^41−44^

Numerical integration techniques, while practical, are not accurate, and more importantly do not easily provide derivatives for the quantities that they compute. This is true for the computations of surface area of a union of balls described above. Analytical alternatives have been proposed, although computing geometric measures of overlapping balls is not an easy task. Approximations have been proposed that treat overlapping balls using a probabilistic model^45−47^ or by fully ignoring them.^48,49^ Such approximations are ideally suited for the parallel architecture of graphics processing units (GPU) .^50^ They remain approximations, however, that are prone to singularities introduced by numerical errors or by discontinuities in the derivatives.^51,52^ Better analytical methods refine the geometric representation of the molecule, considered as a union of pieces of balls.^53−58^

### This Work

To model the nonpolar contribution to the solvation energies of a biomolecular system, in support of the morphometric model described by eq 2, we consider weighted versions of four measures of the geometry of the union of balls, namely, the volume, surface area, integrated mean curvature, and integrated Gaussian curvature, as well as their derivatives with respect to the positions of the ball centers. This paper presents an extension of a large body of work in which these measures and their derivatives have been characterized before in the context of the Voronoi decomposition of a space filling diagram.^59−64^ Its contributions are two-fold. First, we present comprehensive and consistent sets of equations for the expression of the four measures and their derivatives in intrinsic geometry, i.e., as functions of the distances between the centers of the atoms only. Second, we establish parallel algorithms for computing the measures and their derivatives, targeting very large molecular systems with millions of atoms. We use viral capsids to illustrate the performances of these algorithms. Information about the structures of such capsids was recovered from the Protein Data Bank^8^ or from the VIPER database.^65^

### Outline

The next section, Measuring Union of Balls, provides a brief description of the Alpha Shape theory and its application to measuring a union of balls. It includes subsections that provide expressions for the weighted volume, surface area, mean curvature, and Gaussian curvature and their derivatives, all expressed in terms of the distances between the centers of the balls. The explicit constituents for those expressions are provided in the Supporting Information, parts A–D. The following section, Algorithm and Implementation, describes our parallel implementation of this theory. It includes testing on a set of large virus capsids. The last section concludes the paper.

## Measuring Union of Balls

Given a collection of *N* 3-dimensional bodies, *P*_*i*_, any geometric measure of the union of the *P*_*i*_ can be derived from the principle of *inclusion–exclusion*. That is, a measure of the union, ∪_*i*_*P*_*i*_, is expressed as an alternating sum of the measures of the intersections of the *P*_*i*_. To make the inclusion–exclusion formula amenable to computation, however, two issues need to be solved. First, we need to reduce significantly the number of terms it includes, as a brute force application of the formula leads to an algorithm with exponential running time, as the total number of potential intersections of *P*_*i*_ terms is 2^*N*^ – 1. Second, we need analytical formulas for computing the measures of the those intersections of bodies. The next three subsections provide solutions to these two issues when the bodies are 3D balls.

### Background on Voronoi Decompositions and Dual Complexes

**Figure 2:**
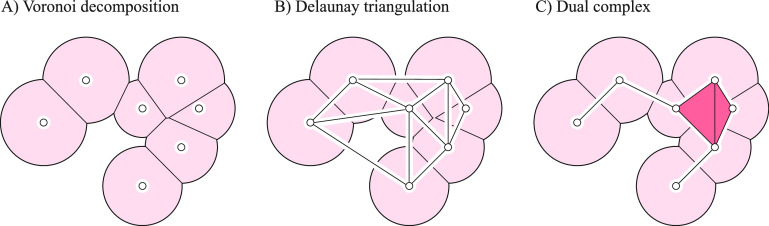
Voronoi decomposition and dual complex of a union of disks. (A) Given a finite set of disks, the Voronoi diagram corresponds to a decomposition of the whole plane into regions, one for each disk, such that any point that belongs to the region corresponding to disk *D*_*i*_ is closer to that disk than to any other disk, with the distance to *D*_*i*_ being the power distance (see text for details). In the graphics, we have restricted the Voronoi diagram to the region covered by the disks. This defines a decomposition of the union of disks into convex regions. (B) The Delaunay triangulation is the dual of the Voronoi diagram that is constructed by defining edges between disk centers of neighboring Voronoi regions. (C) The dual complex is a subset of the Delaunay triangulation, limited to the edges and triangles (dark red) whose corresponding Voronoi regions fully intersect within the union of disks.

Let us consider a finite set of closed balls, *B*_*i*_, with centers *z*_*i*_ and radii *r*_*i*_, and let *S*_*i*_ be the sphere that is the boundary of *B*_*i*_. We define the *power distance* between a point *x* and a ball *B*_*i*_ as . The *Voronoi region* of *B*_*i*_ includes all points *x* that are at least as close to *B*_*i*_ as to any other ball: . It is a convex polyhedron obtained as the common intersection of finitely many closed half-spaces, one per ball *B*_*j*_ ≠ *B*_*i*_. The collection of all Voronoi regions, *V*_*i*_, is the *Voronoi diagram* of the balls. Note that their union covers the entire space. The intersection of the Voronoi diagram with the union of balls *B*_*i*_ decomposes this union into convex regions, as shown in Figure 2A.

The *Delaunay triangulation* is the dual of the Voronoi diagram. It is obtained by defining an edge between the centers of the balls *B*_*i*_ and *B*_*j*_ if and only if the two corresponding Voronoi regions share a common face. In addition, we generate a triangle connecting *z*_*i*_, *z*_*j*_, *z*_*k*_ if *V*_*i*_, *V*_*j*_, *V*_*k*_ intersect in a common line segment, and we generate a tetrahedron connecting *z*_*i*_, *z*_*j*_, *z*_*k*_, *z*_*l*_ if *V*_*i*_, *V*_*j*_, *V*_*k*_, *V*_*l*_ meet at a common point. A 2D version of the Delaunay triangulation is illustrated in Figure 2B. Assuming general positions of the balls, those are the only cases to be considered. We call this the *generic case*. This generic case is rare in practical implementations because of finite precision for the computer representations of the coordinates and radii. It is, however, possible to simulate a perturbation of the union of balls that always restores the generic case.^66^

Next, we limit the construction of Delaunay triangulation to within the union of balls. In other words, we draw a dual edge between the two vertices, *z*_*i*_ and *z*_*j*_, only if *B*_*i*_∩*V*_*i*_ and *B*_*j*_∩*V*_*j*_ share a common face and similarly for triangles and tetrahedra. The result is a subcomplex of the Delaunay triangulation, which is referred to as the *dual complex* of the set of balls (see Figure 2C). Our objective is to use the dual complex X = ∪_*i*_*B*_*i*_, corresponding to a biomolecule to compute its nonpolar solvation free energy, which is expressed in a general form as
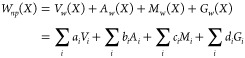
3Here, *V*_*w*_, *A*_*w*_, *M*_*w*_, and *G*_*w*_ are the total weighted volume, weighted surface area, integrated weighted mean curvature, and integrated weighted Gaussian curvature of the union of balls. The *a*_*i*_, *b*_*i*_, *c*_*i*_, and *d*_*i*_ are weights, while the *V*_*i*_, *A*_*i*_, *M*_*i*_, and *G*_*i*_ are the contributions of ball *i* to the total corresponding measure of the union of balls. The sum extends over all atoms of this union. The Voronoi decomposition of the union of balls described above allows us to compute the different terms in these equation based on intersections of up to four balls only.

### Area and Volume Formulas

Write *K* for the dual complex. A simplex, *s*, in *K* can be understood abstractly as a collection of balls: one ball if it is a vertex, two if it is an edge, three if it is a triangle, and four if it is a tetrahedron. As proved in ref (59), the inclusion–exclusion formula that corresponds to the dual complex gives the correct volume and surface area of a union of balls. Let *s*_*i*_ be the vertex corresponding to the ball *B*_*i*_, *s*_*ij*_ the edges of balls *B*_*i*_ and *B*_*j*_, *s*_*ijk*_ the triangle of balls *B*_*i*_, *B*_*j*_, *B*_*k*_, and finally, *s*_*ijkl*_ the tetrahedron of four balls, *B*_*i*_, *B*_*j*_, *B*_*k*_, and *B*_*l*_. Then:

**Proposition 1: (Area)**
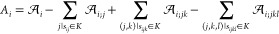
4
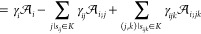
5

**Proposition 2: (Volume)**
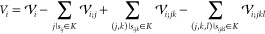
6
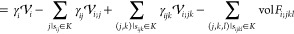
7Here,  is the volume of the ball *B*_*i*_,  is the contribution of *B*_*i*_ to the volume of the intersection of the balls *B*_*i*_ and *B*_*j*_, etc. Similar definitions are used for the surface areas .

Note that even though the eqs 4 and 6 for the surface area and volume are minimal as they only consider up to four levels in the inclusion–exclusion formula, it is possible to find even shorter expressions if noninteger coefficients are considered. Those expressions correspond to the short inclusion–exclusion method; it is described in detail in ref (59). In this method, the areas and volumes are expressed as the sums of the contributions of intersections of at most three balls, with angular coefficients γ_*i*_, γ_*ij*_, and γ_*ijk*_, with the exception of the term vol *F*_*i*;*jkl*_ (a fraction of the Voronoi region of *B*_*i*_; see Supporting Information, part A). These coefficients γ are the normalized exposed angles of the simplices;^60^ they integrate the contributions of the tetrahedra of the dual complex. For vertices and edges, these angles can be expressed as fractions of solid and dihedral angles inside tetrahedra. If we define Ω_*i*;*jkl*_ as the solid angle at vertex *z*_*i*_ and ϕ_*ij*;*kl*_ as the dihedral angle at the edge *z*_*i*_*z*_*j*_ in the tetrahedron defined by *z*_*i*_, *z*_*j*_, *z*_*k*_, *z*_*l*_, the coefficients are
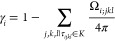
8
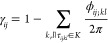
9
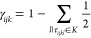
10Expressions for the derivatives with respect to the Cartesian coordinates of the centers of the balls are available for the surface area^61^ and for the volume.^60^ Alternate expressions are available for the same derivatives with respect to the distances between the center of these balls.^67^ Note that these distances define internal coordinates for the system, which are invariant under rigid body transformations (rotations and translations). We recall those derivatives here:

**Proposition 3: (Area derivative)**
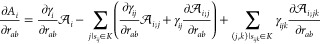
11

**Proposition 4: (Volume derivative)**
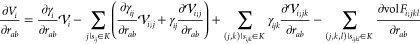
12

The derivatives of the surface area and volume are expressed in eqs 11 and 12, respectively. They are derived from the corresponding simplified, angle-weighted inclusion–exclusion eqs 5 and 7, respectively. Note that there are no derivatives of  and , which are constant, and that there are no terms involving the derivatives of γ_*ijk*_: these derivatives are piecewise zero because the γ_*ijk*_ are piecewise constant. Their values change at nongeneric states, where their derivatives are not defined.^60,61^ Finally, we note that the derivatives of *A*_*i*_ and *V*_*i*_ with respect to the distance *r*_*ab*_ between the centers *z*_*a*_ and *z*_*b*_ of the two balls *B*_*a*_ and *B*_*b*_ are nonzero if and only if *i*, *a*, and *b* belong to a simplex of *K*.

Proofs of eqs 4, 5, 6, 7, 11, and 12 and additional formulas are provided in refs (59−61 and 67). We summarize them in Supporting Information, part A, for sake of completeness.

### Mean Curvature formulas

Akopyan and Edelsbrunner recently derived theorems for computing the integrated mean curvature over the surface of a union of balls using the dual complex.^63^ They distinguish between two terms: the contribution of the spherical patches and the contribution of the accessible circular arcs at the intersections of two spheres. Along these circular arcs, the mean curvature is partitioned equally between the two spheres involved. This leads to the following formula for the mean curvature and its derivatives in terms of the edge lengths in the dual complex:

**Proposition 5: (Mean curvature)**
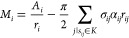
13

**Proposition 6: (Mean curvature derivative)**
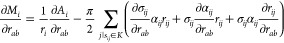
14

**Figure 3:**
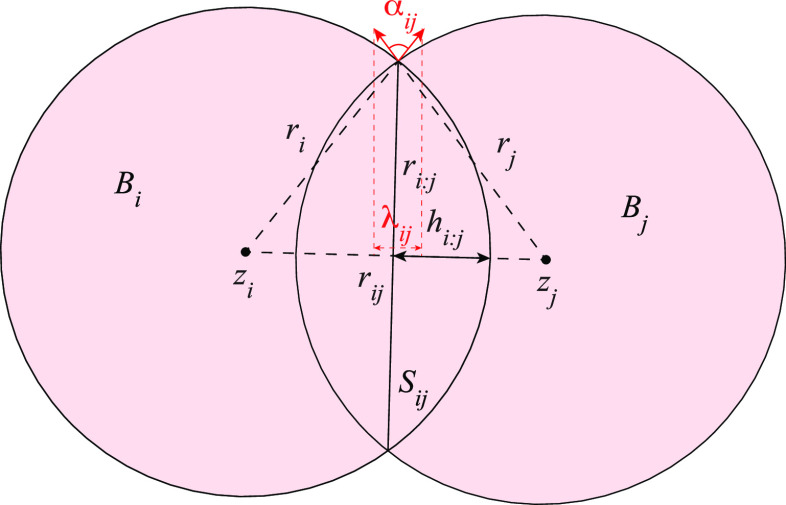
Intersection of two disks.

In addition to the contribution *A*_*i*_ of the sphere *S*_*i*_ to the total surface area of the union of balls, these equations involve three new terms, *r*_*ij*_, α_*ij*_, σ_*ij*_, all associated with two balls *B*_*i*_ and *B*_*j*_ that form a simplex of the dual complex. The spheres *S*_*i*_ and *S*_*j*_ that bound those balls intersect at a circle *S*_*ij*_; *r*_*ij*_ is the radius of this circle, and α_*ij*_ is the angle between the unit normals of the spheres at any point of *S*_*ij*_; see Figure 3. Finally, σ_*ij*_ is the fraction of the length of *S*_*ij*_ that is at the boundary of the union of balls (i.e., not covered by other balls). Akopyan and Edelsbrunner^63^ established formulas for these three terms as functions of the Cartesian coordinates of the centers of the balls in the union. In Supporting Information, part B, we revisit these formulas using internal coordinates (namely, the distances between the centers of the balls) instead.

### Gaussian Curvature Formulas

In parallel to the mean curvature formula, Akopyan and Edelsbrunner established a formula for the Gaussian curvature that distinguishes between three terms: the contribution of the spherical patches, the contribution of the circular arcs at the intersections of two spheres, and the contribution of the accessible corners at the intersection of three spheres.^64^ This leads to the following formula for the Gaussian curvature and its derivatives in terms of edge lengths in the dual complex:

**Proposition 7: (Gaussian curvature)**
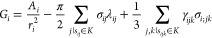
15

**Proposition 8: (Gaussian curvature derivative)**
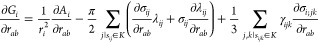
16

All variables have been defined above, except for λ_*ij*_ and σ_*i*;*jk*_, associated with two and three spheres, respectively. Two spheres, *S*_*i*_ and *S*_*j*_, with centers *z*_*i*_ and *z*_*j*_ that form an edge in *K* intersect at a circle *S*_*ij*_. λ_*ij*_ is the combined length of the unit normals of the spheres at any point of *S*_*ij*_ after projection on the line passing through *z*_*i*_ and *z*_*j*_; see Figure 3. Three spheres, *S*_*i*_, *S*_*j*_, and *S*_*k*_, that form a triangle in *K* intersect in two points, *P*_*ijk*_ and *P*_*ikj*_. σ_*ijk*_ is the fraction assigned to *i* of the solid angle spanned by the unit normals of *S*_*i*_, *S*_*j*_, and *S*_*k*_ at one of those points. Akopyan and Edelsbrunner^64^ established formulas for those two terms. In Supporting Information, part C, we revisit those formulas using internal coordinates.

### The Nonpolar Solvation Free Energy *W*_*np*_

Recall that the nonpolar contribution to the solvation free energy of a union of balls, *X*, is
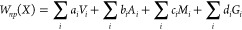
17in which *V*_*i*_, *A*_*i*_, *M*_*i*_, and *G*_*i*_ are the contributions of ball *i* to the total volume, surface area, integrated mean curvature, integrated Gaussian curvature of *X*, respectively, and *a*_*i*_, *b*_*i*_, *c*_*i*_, and *d*_*i*_ are the coefficients corresponding to pressure, surface tension, and bending rigidities. In the previous subsections, we have established formulas for those contributions, as well as for their derivatives with respect to internal coordinates. For any given pair of balls, *B*_*a*_ and *B*_*b*_, that belong to the dual complex, *K*, of *X*, we have
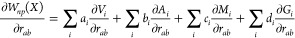
18in which , , ,  are given by eqs 12, 11, 14, and 16, respectively, with all details given in the Supporting Information. Once the derivatives in terms of internal coordinates are available, derivatives with respect to Cartesian coordinates are easily computed using the chain rule:

**Proposition 9: (Derivative of***W*_***np***_**)** The gradient **a** of the nonpolar solvation free energy is
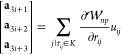
19in which *u*_*ij*_ = (*z*_*i*_ – *z*_*j*_)/*r*_*ij*_ is the unit vector along the edge from *z*_*j*_ to *z*_*i*_.

## Algorithm and Implementation

Our software for computing geometric measures of biomolecules has gone through successive revisions. AlphaVol was our original software package, which implemented the Alpha Shape theory for the volumes and surface areas of biomolecules;^62^ its origins can be traced to the Alpha Shape package.^68^ AlphaVol was partially redesigned into a new package, UnionBall, with modification needed to deal with large molecular systems.^67^ We have now completely redesigned UnionBall into a new package, AlphaMol, written in C++. AlphaMol implements all four intrinsic volumes, as well as their derivatives with respect to atomic coordinates. Each of these measures is possibly weighted, i.e., the contribution of each atom is weighted by a constant provided as input to the software, with a different constant for each of the intrinsic volumes. AlphaMol takes as input a set of balls in , each specified by the coordinates of its center and the radius, as well as by its four weights. In the case of biomolecules, the coordinates of the center of the balls are extracted from the corresponding PDB file, while the radii are defined according to the chemical nature of the atoms, using one of several standard sets of radii (in the following we use the OPLS force field^69^). These radii may be enlarged by the radius of a water probe (usually 1.4 Å), should the measures correspond to the accessible surface of the molecule. The algorithm includes three steps:

Step 1. Build the Delaunay triangulation.

Step 2. Extract the dual complex from the Delaunay triangulation.

Step 3. Compute the geometric measures of the union of balls using the dual complex.

Just like AlphaVol and UnionBall, AlphaMol uses standard algorithms from computational geometry for the first two tasks.^70,71^ We have designed our own algorithm for measuring the union (step 3) .^60,61,63,64^ We have made modifications to these algorithms compared to AlphaVol and UnionBall, as our interests are mostly measuring biomolecules, with a focus on scalability, namely, the ability to deal with very large biomolecules. We describe those modifications in the following subsections, for the sequential and parallel version of AlphaMol, respectively.

### A Sequential Algorithm for Measuring Biomolecules

We implemented the randomized incremental algorithm from Edelsbrunner and Shah^70^ to construct the Delaunay triangulation of a union of balls. In this algorithm, the triangulation is built incrementally, by adding one ball at a time. The input balls are preprocessed with a random permutation. Four dummy balls whose centers lie at infinity are added so that all input balls are contained in the tetrahedron they define. Let *DT*_*i*_ be the Delaunay triangulation at step *i* of the construction (*DT*_*i*_ contains the four balls at infinity as well as *B*_1_, *B*_2_, ..., *B*_*i*_). The algorithm proceeds by iterating three steps:

for *i* = 1 to *N* do
(1) Identify the tetrahedron *t* ∈ *DT*_*i*–1_ that contains *z*_*i*_.
(2) Add *z*_*i*_ as a vertex and decompose *t* into four tetrahedra.
(3) Flip locally all non-Delaunay triangles attached to *z*_*i*_.
endfor.

The randomization guarantees a theoretical expected running time of *O*(*N* log  *N*) with an additional linear term in the number of simplices in the Delaunay triangulation.^70^ In , the number of simplices can be as large as a constant times *N*^2^. However, for well-packed data—which is typical for biomolecules—this number is at most a constant times *N*, leading to an expected running time of *O*(*N* log  *N*).

In practice, a different behavior is observed for very large molecules (such as macromolecular assemblies with millions of atoms, for example virus capsids). This is unfortunately a known problem. Virtual memory operating systems cache recently used data in memory, under the assumption that they are more likely to be used again soon. If the new ball to be inserted in step 1 is not included in one of the recent tetrahedra, the cache will not be useful. This scenario is likely if the order in which the balls are inserted is random. A possible solution is to create 3D locality, by ordering the balls first such that a ball at position *i* is mostly local with respect to the previous balls in the ordering. Interestingly, the order in which atoms are stored in a PDB file is inherently local.^67^ UnionBall implemented this idea. Instead of randomizing the balls, as in AlphaVol, it keeps the ordering provided by the input PDB file. The effect was significant: UnionBall is substantially faster than AlphaVol, especially for large molecular systems.^67^ With this simple trick, however, there are no guarantee for the expected running time.

Amenta, Choi, and Rote^72^ developed a scheme for ordering points before computing the Delaunay triangulation that maintains enough randomness so that the theoretical complexity of the algorithm is conserved. Their Biased Randomized Insertion Order (BRIO) method was shown to significantly improve the running time of Delaunay triangulation for large number of points.^72^ Later, Liu and Snoeyink^73^ proposed a different method for ordering the points based on the Hilbert curve. They showed that reordering points using such a Hilbert curve significantly sped up the point location in step 1 of the Delaunay triangulation algorithm.^73^

**Figure 4:**
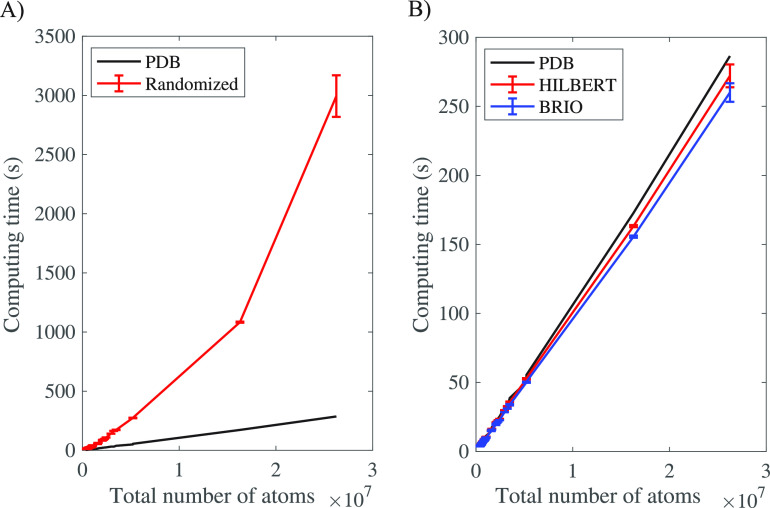
(A) The running times of AlphaMol for measuring biomolecules (the 68 virus capsids in our database; see Supporting Information, part E), when the atoms are inserted randomly (red line) or based on the order provided by the PDB file (black line). (B) Comparing the running times of AlphaMol when atoms are inserted based on the PDB order (black line), or randomly inserted, followed by ordering based on the Hilbert curve (red line) or followed by BRIO-Hilbert ordering (blue curve). In both (A) and (B), we show the mean and standard deviation over five random trials. Computations were performed on a single core on an iMac computer with an 3.8 GHz 8-core 10th-generation Intel Core i7.

We implemented our own version of BRIO in AlphaMol as an option. BRIO proceeds by organizing the points randomly into *rounds*, using a logarithmic scheme.^72^ Within each round, points can be inserted into any order, allowing for locality. In the original BRIO, within a round, the points were ordered using a *kd*-tree; we used the Hilbert curve instead. We added another option to AlphaMol, in which the ordering follows the Hilbert curve directly (this is equivalent to BRIO with a single round). We compared our versions of BRIO and of the Hilbert curve ordering with the predefined ordering imposed by the PDB file (as implemented in UnionBall) and with randomized ordering (as implemented in AlphaVol) on a set of 68 virus capsids (see Supporting Information, part E, for a full list). These virus capsids vary in size from 400,000 atoms to 26,000,000 atoms, representing a broad range of sizes for large biomolecular systems. Results of the comparisons of the different ordering schemes are illustrated in Figure 4.

**Figure 5:**
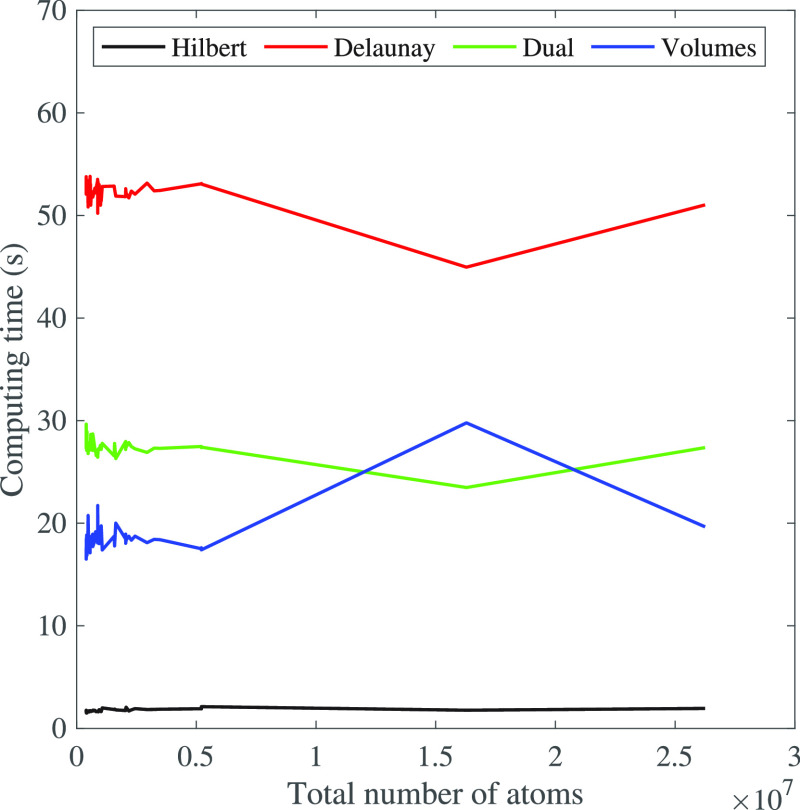
Fraction of running times of the different components of AlphaMol for measuring the 68 virus capsids in our database; (see Supporting Information, part E), in percentage: black, Hilbert curve ordering of the atoms (average 1.7%); red, weighted Delaunay triangulation of the ball centers (average 52.4%); green, filtering the Delaunay triangulation to generate the dual complex (average 27.4%); and blue, computing the intrinsic volumes based on the dual complex (average 18.5%).

As illustrated in Figure 4A, not randomizing the order in which points are inserted resulted in a significant improvement in performance. This was already observed with UnionBall.^67^ Removing the randomization leads to an observed linear dependence of the running time on the number of weighted spheres considered. Randomization followed by ordering based on a spacing-filling curve, or on our modified BRIO method, leads to further speed-up, albeit small; see Figure 4B. Note that the differences in running time between the Hilbert curve ordering and BRIO are not significant. In the following, we use the Hilbert curve ordering when necessary. We note that both BRIO and the Hilbert curve method require preprocessing of the data that comes with its own computational cost. This cost, however, is minimal, representing 1.7% of the total computing time, on average; see Figure 5. The bulk of the calculation comes from computing the Delaunay triangulation (52.4% on average), followed by the extraction of the dual complex (27.4%), and the computations of the intrinsic volumes and their derivatives (18.5%).

### A Parallel Algorithm for Measuring Biomolecules

As described above, measuring a biomolecule represented by a union of balls involves three steps: computing the Delaunay triangulation of the centers of the balls weighted by their radii, filtering the simplices of the Delaunay triangulation to build the dual complex, and building the inclusion–exclusion formulas for the intrinsic volumes of the union of balls. Of these three steps, the second and the third can be easily parallelized, as they basically involve iterating over simplices. Unfortunately, parallelizing the computation of the Delaunay triangulation is a difficult task that remains a hot topic in research.^74−80^ Considering that computing the Delaunay triangulation is more than 50% of the total computing cost of AlphaMol (see Figure 5), this is a concern.

**Figure 6:**
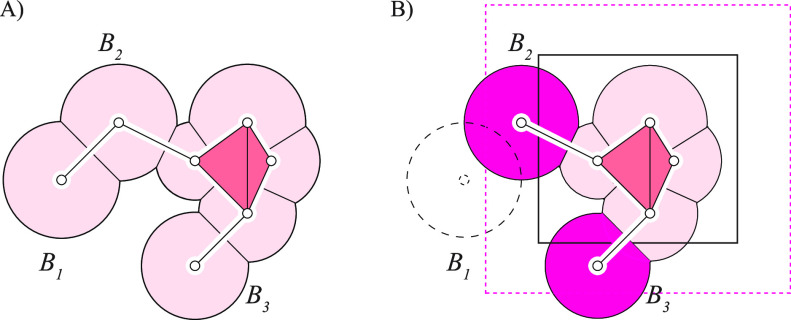
Splitting the computation of the intrinsic volumes of a union of balls. Let us consider the union of balls represented in panel A (this is the same union as in Figure 2, in which we show the dual complex overlaid with the restriction of the Voronoi diagram to within the portion of the plane covered by the balls). In panel B, we limit this union to those balls whose centers belong to the rectangular block shown with solid sides; those balls are colored rose. We expand this block with a buffer zone, delimited by the dashed, magenta sides. Balls *B*_2_ and *B*_3_, whose centers are within this buffer zone, interact with balls from the rectangular block. All other balls (here *B*_1_) are ignored. Applying AlphaMol to the balls in the block and in the buffer zone leads to the correct computation of the intrinsic volumes for the balls in the block. The procedure is repeated in parallel for all blocks covering the union.

**Figure 7:**
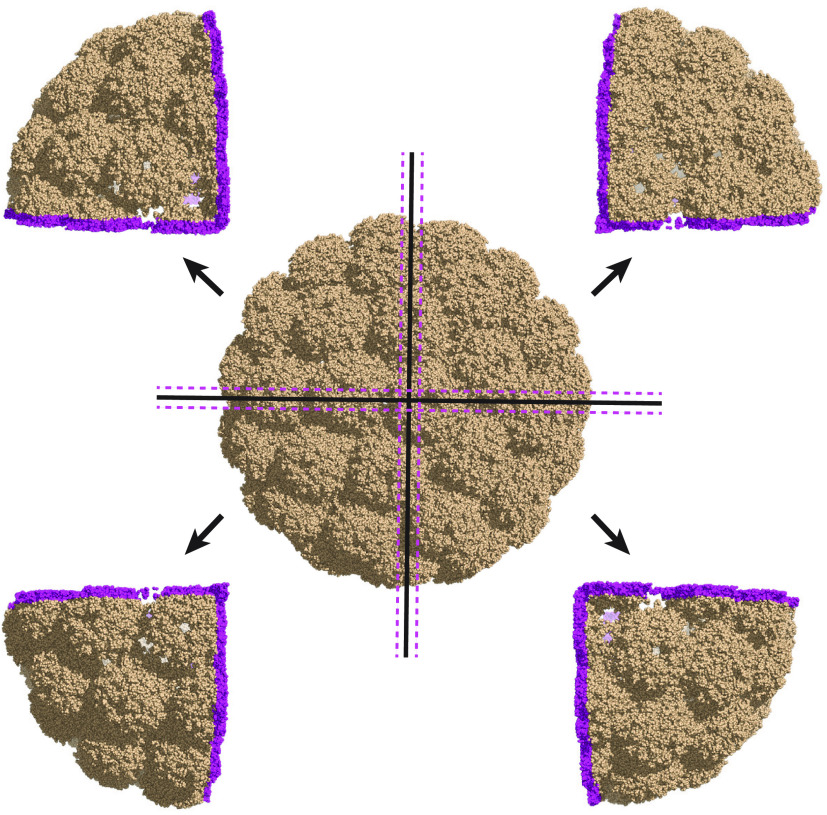
Splitting the computation of the intrinsic volumes of the murine polyomavirus (PDB code 1sid) over four processors. The 1,020,180 atoms are divided into four partitions of approximately equal size, using a *kd*-tree algorithm. Each partition correspond to a rectangular block. Each block is complemented with a buffer zone, such that atoms in this buffer zone (shown in *magenta*) may interact with the atoms of the block. Each block together with its buffer zone is assigned to one processor, which then runs the full AlphaMol algorithm. As each block with buffer zone includes approximately one-quarter of the atoms, and the computations on the blocks are run in parallel, it is expected that the total computation time be reduced by a factor of 4.

Many parallel Delaunay triangulation algorithms have been proposed. Most focus on partitioning the domain that contains the points so that each partition can be triangulated separately and in parallel with the others.^74−80^ The bottleneck of this approach, however, is the merging of the triangulations from the different partitions to generate the complete triangulation.^76^ The tetrahedra at the borders of a partition have to be connected to tetrahedra in adjacent partitions, often leading to local retriangulations. This merging step is sometimes referred to as *stitching* and is known to be difficult. We note, however, that the goal of AlphaMol is to compute the contributions of all atoms to the intrinsic volumes of the biomolecule of interest and not generate the overall Delaunay triangulation. We propose a different parallel strategy for computing these contributions, that still uses the concept of partitioning the whole domain, but with a focus on the volumes, and not the Delaunay triangulation. It is explained in Figure 6 and illustrated for a virus capsid in Figure 7.

Briefly, we partition all atoms of the biomolecule into 3D rectangular blocks, using a *kd*-tree algorithm. Our goal is to apply the sequential algorithm of AlphaMol to each block, using a different processor for each block. We note, however, that atoms at the edges of a block may interact with atoms from neighboring blocks. If those atoms are not included in the calculation, the intrinsic volumes found for the atoms in the block will be inexact. We therefore expand each block with a buffer zone to include the atoms neighboring the block. The width of this buffer zone is set to 2*R*_max_, in which *R*_max_ is the radius of the largest ball in the union of balls representing the biomolecule. This value ensures that all atoms that potentially interact with the atoms in the block will be accounted for. We then apply the AlphaMol algorithm to all atoms in the block and in its buffer zone. We finally retain the intrinsic volumes of the atoms in the block. The choice of the buffer zone guarantees that simplices sharing a vertex in the block are exactly the simplices sharing this vertex in the complete Delaunay triangulation, a property that does not hold for the vertices in the buffer zone. It follows that the intrinsic volumes of the balls whose centers lie in the block are computed correctly, while those of the balls whose centers lie in the buffer zone may be incorrect. Each block and its buffer is dealt with on a different processor, and as the partitioning of the atoms is balanced, we expect a speed-up proportional to the number of blocks.

**Figure 8:**
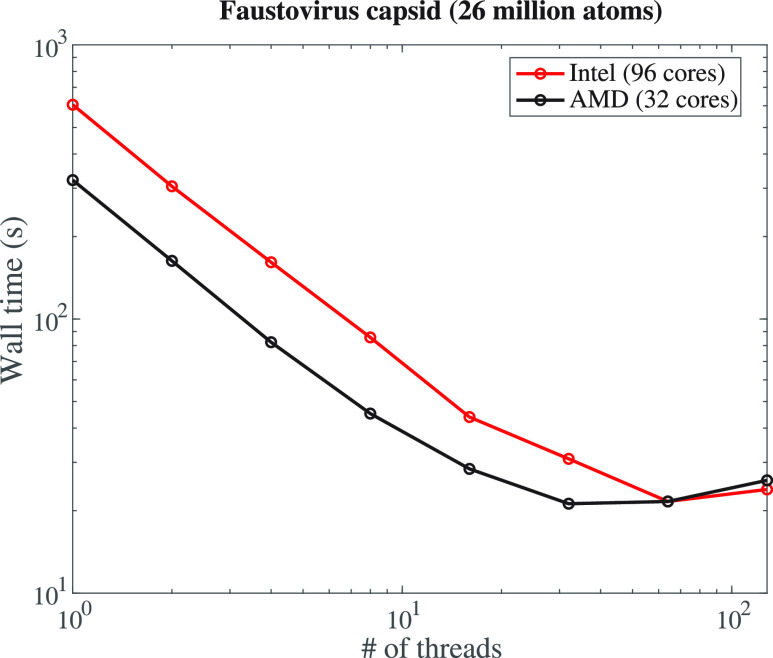
Execution (wall) time of AlphaMol as a function of the number threads requested, for computing the intrinsic volumes and their derivatives of the capsid of the faustovirus (PDB code 5j7v), which consists of approximately 26 million atoms. Computations were performed on an Intel Xeon processor at 2.70 GHz, with 96 cores (192 threads) (red), or an AMD Threadripper processor at 2.2 GHz, with 32 cores (64 threads).

Figure 8 shows the wall time for the execution of AlphaMol on the capsid of faustovirus (PDB code 5j7z,^81^ 26 million atoms), with different numbers of threads requested by the software. The virus capsid is divided into *m* partitions based on a *kd*-tree, and each partition is handled separately on a different thread. Computations were performed on two different multicore computers, one with an Intel Xeon multicore CPUs running at 2.70 GHz, with 96 cores (192 threads), and a second with an AMD Threadripper multicore CPUs running at 2.2 GHz, with 32 cores (64 threads). The AMD computer is more recent than the Intel computer. As expected, we observe a significant speed-up when AlphaMol is run on multiple threads. The gain in time is significant. It takes 605 s with a single Intel Xeon thread and 321 s with a single AMD thread to compute the weighted volumes and their derivatives for the capsid of faustovirus. In comparison, it takes 161 or 82.3 s on four Intel threads or four AMD threads, respectively, and it only takes 30.9 or 21.2 s to do the same calculation using 32 Intel threads or 32 AMD threads, respectively. The speed-up is nearly linear between 1 and 32 threads for both types of processors. We note, however, that the speed-up is marginal between 32 threads and 64 threads for the AMD processor and between 64 threads and 128 threads for the Intel processor (see Figure 8). We believe that this behavior is reflective of the computer architecture, and not of the software itself. The AMD processor has 64 threads, but only 32 physical cores. We do not expect that two threads on a single core will be significantly faster than a single thread on that core, as those two threads share the same resources. The Intel processor has 96 physical cores. However, those cores are spread equally over four sockets, each with 24 cores. As such, we also expect a slow down in speed-up for large numbers of threads that need to share the same memory resources.

**Figure 9:**
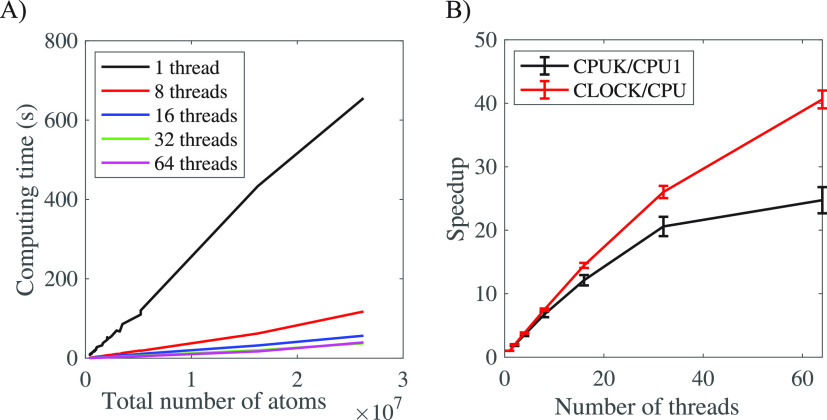
(A) Execution (wall) time of AlphaMol as a function of the number of atoms for different numbers of processors. (B) The speedup (computed as the ratio of total CPU time over wall time) is plotted against the number of threads used by the program. Computations were performed on an Intel Xeon processor at 2.70 GHz, with 96 cores (192 threads).

**Figure 10:**
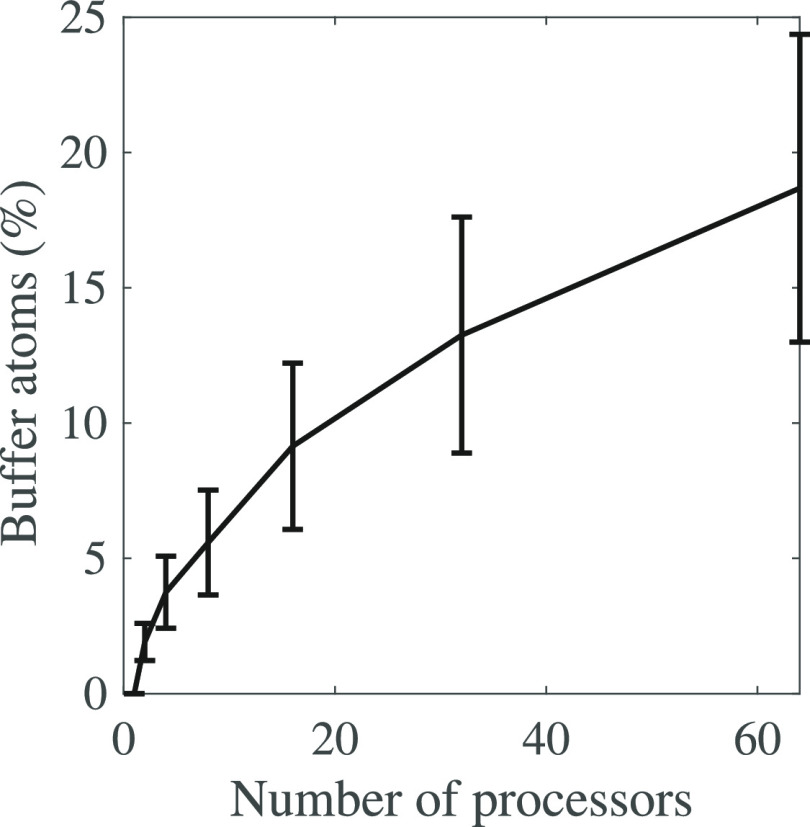
Percentages of atoms in the buffer zones as a fraction of the total number of atoms for the parallel version of AlphaMol as a function of the number of processors. An atom is said to belong to the buffer zones if it belongs to at least one of the buffer zones. The percentage increases with the number of processors, leading to progressively more redundant computations that ultimately limit the impact of using multiple processors.

To reduce the risk that our conclusions are anecdotal, we repeated the calculations on all 68 virus capsids in our database. We only used the Intel computer, but found similar results with the AMD computer. Figure 9 shows the wall time as well as the speed-up for the execution of AlphaMol on the virus capsids, with different numbers of threads. Just like for the faustovirus, we observe a significant speed-up when multiple threads are added. This speed-up is nearly linear up to 32 cores, and then slows down. There are two reasons for the slow down in speed-up as more threads are added, namely, the architecture of the computer and progressively more redundant computations for larger numbers of threads. To illustrate the latter, we use two ways to calculate the speed-up. First, we compare the wall time with the total CPU time, represented as CLOCK/CPU. Based on this notion, the speed-up is nearly linear up to 32 processors, and slows down thereafter, still reaching 40 for 64 processors. It shows that there is little communication between the master processor and the at least 32 additional processors, indicating that the parallelism is effective. Second, we compare the wall time when running on *k* processors, with the running time from a serial (i.e., one processor) independent calculation. Based on this second notion, the speed-up is less effective, with 64 processors not being significantly more effective than 32 processors. The two notions should give similar results if the splitting is balanced so that each processor deals with about *N*/*k* atoms, *N* being the total number of atoms in the biomolecule. This is not the case, however, as the algorithm adds a buffer zone to each partition to make sure that the computation is correct. Each atom in the buffer zones will be considered at least twice, since it also belongs to one of the partitions. As expected, the number of atoms treated at least twice increases with the number of processors; see Figure 10. This leads to redundant computations whose importance increases when the number of processors increases, ultimately leading to a plateau in the speed-up brought by the parallelism. It remains that we do reach a significant speed-up through this parallelism, with an average factor of 20-folds with 32 processors.

## Conclusion

The Alpha Shape theory provides an accurate and robust method for computing the geometric measures of a biomolecule.^59−64^ Among these measures, the intrinsic volumes are used to quantify the interaction between a biomolecule and surrounding water in the so-called morphometric model.^33−35^ Several implementations of the Alpha Shape theory for measuring biomolecules exist, including our own, AlphaVol^62^ and UnionBall.^67^ These implementations, however, were limited to computing the volumes and surface areas of biomolecules.

In this paper, we have derived homogeneous formulas for the expressions of all four intrinsic volumes and their derivatives and implement them into a new package, AlphaMol. The only variables in these formulas are the interatomic distances, making them insensitive to translations and rotations. Recent spectacular advances in structural biology have produced an abundance of data on large macromolecular complexes, such as full size virus capsids^82^ that contain several millions of atoms. Modeling these large systems is as important as modeling smaller proteins or nucleic acids; see for example the simulations of the HIV capsid that include over 60 million atoms.^17^ To make sure that AlphaMol remains practical, we have adapted its underlying algorithms in two ways. First, we have included an ordering scheme based on Hilbert curves to improve the localities of the atoms as they are introduced sequentially for generating the Delaunay triangulation of the atom centers, which is at the core of the Alpha Shape theory. Second, we have introduced a parallel version of AlphaMol, which partitions the atoms of the biomolecule of interest into 3D rectangular blocks, using a *kd*-tree algorithm. We then apply the sequential algorithm of AlphaMol to each block, augmented by a buffer zone to account for atoms that may overlap with atoms in the block. The presence of the buffer zones, however, leads to redundant computations that ultimately limit the impact of using multiple processors. In our current version, 32 processors led to a significant speed-up (20 times on average for 68 virus capsids ranging from 400,000 atoms to 26 million atoms), with marginal improvements for a higher number of processors.

Ultimately, we would like to push the parallelism to hundreds of processors, such as those that are available on a GPU. Recently, there was an attempt to do so for computing the Alpha Shape of a molecule,^83^ using a mixed CPU-GPU algorithm showing speed-ups in the order of 25, which is similar to what we observed for multiple CPUs. We do see, however, some roadblocks for a GPU-only implementation of AlphaMol. To our knowledge, all current GPU implementations of the 3D Delaunay triangulation compute a near-Delaunay structure on the GPU, followed by transformations on the CPU to generate a valid Delaunay triangulation.^84^ In addition, computing a valid Delaunay triangulation requires robust geometric predicates to account for possible degeneracies. In our implementation, we rely on the Simulation of Simplicity (SoS) to remove those degeneracies.^66^ The SoS method is based or arbitrary precision arithmetics. There are currently only limited GPU implementations of arbitrary precision arithmetics, and they are still under development. Developing a GPU-based computation of intrinsic volumes of biomolecules remains, however, a priority for molecular simulations of very large biomolecular systems.

## Data Availability

All PDB files for the virus capsid structures are available from the Protein Data Bank, http://www.rcsb.org. The sequential version of AlphaMol is available as OpenSource software on github (https://github.com/pkoehl/AlphaMol).

## Supporting Information Available

Proofs of the propositions in main text of the document: surface areas and volumes of ball intersections and derivatives; integrated mean curvature and derivatives; integrated Gaussian curvature and derivatives; and geometry of a tetrahedron. Table S.1: Database of virus capsid structures that are used in this study. (PDF)
