# Supplementary material for: Computing the Volume, Surface Area, Mean, and Gaussian Curvatures of Molecules and Their Derivatives
Source: J Chem Inf Model. 2023 Jan 13;63(3):973–85. doi: 10.1021/acs.jcim.2c01346 (PMC9930125; doi:10.1021/acs.jcim.2c01346)
Supplement: Supplementary file 1 — ci2c01346_si_001.pdf [file ci2c01346_si_001.pdf]

# Supplemental Information for "Computing the Volume, Surface Area, Mean and Gaussian Curvatures of Molecules and Their Derivatives"

Patrice Koehl,[*,†] Arseniy Akopyan,[*,‡] and Herbert Edelsbrunner[*,¶]

†*Department of Computer Science, University of California, Davis, CA 95616, USA*

‡*FORA Capital, Miami, Fl 33131, USA*

¶*IST Austria, 3400 Klosterneuburg, Austria*

E-mail: koehl@cs.ucdavis.edu; akopjan@gmail.com; edels@ist.ac.at

# A. Surface Areas and Volumes of Ball Intersections, and Derivatives

Several formulas have been presented for the volume and surface areas of the intersection of two, three, and four spheres with unequal radii; see for example.[1–3] Here we describe versions of these formulas that depend only on the radii of the spheres and the distance between their centers. Derivations of these versions were originally given in Mach and Koehl, 2011;[4] they are provided here for sake of completeness, as well as a support to provide the derivatives of these geometric measures with respect to the distance between the sphere centers.

In the following, we write $B_i, B_j, B_k, B_l$ be four balls with bounding spheres $S_i, S_j, S_k, S_l$, centers $z_i, z_j, z_k, z_l$, and radii $r_i, r_j, r_k, r_l$.

## Intersection of two balls

The intersection between the two balls $B_i$ and $B_j$ is the union of two caps, as illustrated in Figure 3 in the main paper, which we redraw here as it will be referred to many times below: The inter-

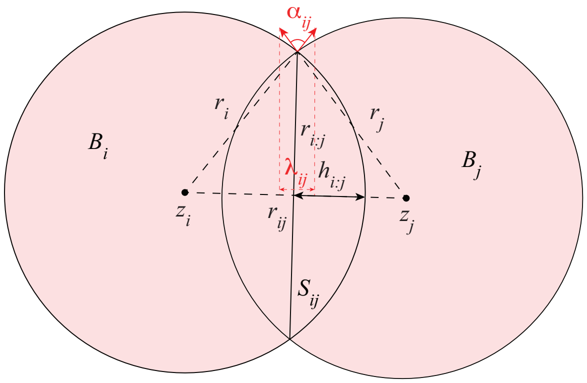


Figure S.1: Intersection of two disks.

section of the two caps is a disk with center $y_{ij}$ and radius $r_{ij} = \|z_i - z_j\|$. We also define the *height* of cap from $S_i$, which we denote $h_{i;j}$. As noted in Bryant *et al.* 2004,[5] the signed distance

between $z_i$ and the plane that contains the disk is:

$$\zeta_i = \frac{r_{ij}}{2} + \frac{r_i^2 - r_j^2}{2r_{ij}}. \tag{S.1}$$

Hence,

$$\begin{aligned} h_{i;j} &= r_i - \zeta_i, && \text{(S.2)} \\ r_{ij} &= \sqrt{r_i^2 - \zeta_i^2}. && \text{(S.3)} \end{aligned}$$

Setting

$$\lambda_{i;j} = -\frac{\partial \zeta_i}{\partial r_{ij}} = -\frac{1}{2} + \frac{r_i^2 - r_j^2}{2r_{ij}^2}, \tag{S.4}$$

we get

$$\begin{aligned} \frac{\partial h_{i;j}}{\partial r_{ij}} &= \lambda_{i;j}, && \text{(S.5)} \\ \frac{\partial r_{ij}}{\partial r_{ij}} &= \frac{\zeta_i \lambda_{i;j}}{r_{ij}}. && \text{(S.6)} \end{aligned}$$

**Proposition S.1:** The intersection between two balls is illustrated in Figure 3 in the main paper. We have:

$$\begin{aligned} \mathcal{A}_{i;j} &= 2\pi r_i h_{i;j}, && \text{(S.7)} \\ \frac{\partial \mathcal{A}_{i;j}}{\partial r_{ij}} &= 2\pi r_i \lambda_{i;j}, && \text{(S.8)} \\ \mathcal{V}_{i;j} &= \frac{1}{3}\pi h_{i;j}^2 (3r_i - h_{i;j}), && \text{(S.9)} \\ \frac{\partial \mathcal{V}_{i;j}}{\partial r_{ij}} &= \pi h_{i;j} \lambda_i (2r_i - h_{i;j}). && \text{(S.10)} \end{aligned}$$

See Bryant *et al.*, 2004[5] for the proof. Note that Equation (S.7) is simply Archimedes' area formula.

## Intersection of three balls

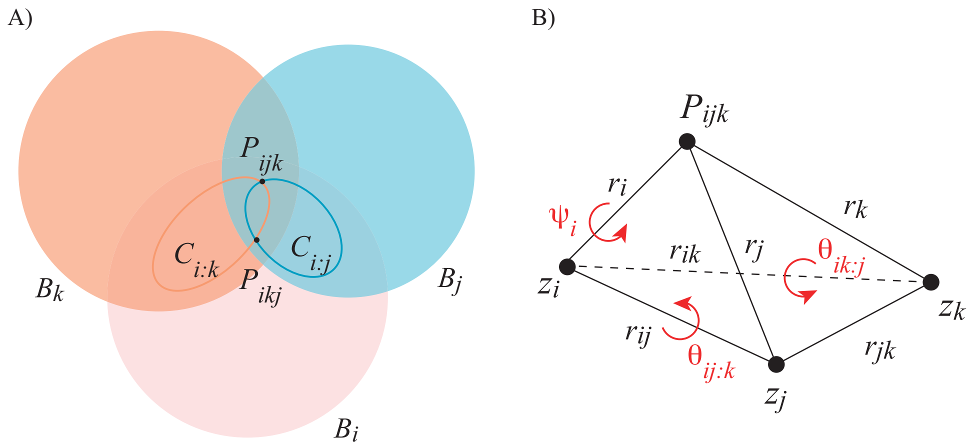


Figure S.2: **A**. Intersection of three balls. **B**. The tetrahedron, $T$, used to measure the intersection of the three balls. $z_i, z_j, z_k$ are the centers of the spheres, and $P_{i;jk}$ is one of the two points common to all three spheres.

The contribution of $B_i$ to the volume and surface area of the intersection of $B_i$, $B_j$, $B_k$ is defined by the intersection of the caps $\mathcal{C}_{i;j}$ and $\mathcal{C}_{i;k}$, illustrated in orange and blue in panel A of Figure S.2. The three spheres, $S_i, S_j, S_k$, intersect in two points, $P_{i;jk}$ and $P_{i;kj}$. We consider the tetrahedron, $T$, whose vertices are the centers of the three balls and the point $P_{i;jk}$; see panel B of Figure S.2. The dihedral angles at the edges $z_iz_j$ and $z_iz_k$ are denoted $\theta_{ij;k}$ and $\theta_{ik;j}$, respectively, while $\psi_i$ is the dihedral angle at the edge $z_iP_{ijk}$.

**Proposition S.2:** The contributions of $S_i$ and $B_i$ to the surface area and volume of the triple intersection are

$$\mathcal{A}_{i;jk} = 2r_i(h_{i;j}\theta_{ij;k} + h_{i;k}\theta_{ik;j} - r_i(\theta_{ij;k} + \theta_{ik;j} + \psi_i - \pi)), \quad \text{(S.11)}$$

$$\begin{aligned}\mathcal{V}_{i;jk} &= \frac{1}{3}r_i\mathcal{A}_{i;jk} - \frac{1}{3}(r_i - h_{i;j})(2r_ih_{i;j} - h_{i;j}^2)(\theta_{ij;k} - \sin\theta_{ij;k}\cos\theta_{ij;k}) \\ &\quad - \frac{1}{3}(r_i - h_{i;k})(2r_ih_{i;k} - h_{i;k}^2)(\theta_{ik;j} - \sin\theta_{ik;j}\cos\theta_{ik;j})\end{aligned} \quad \text{(S.12)}$$

where the dihedral angles are computed from the edge lengths of the tetrahedron $T$; see section D below. Formulas for the contributions of $B_j$ and $B_k$ to the intersection are easily deduced by

index permutation on these equations. The proof of Proposition A.2 can be found in Appendix A of Mach and Koehl, 2011[4] (Lemma 2).

Formulas for the derivatives with respect to edge lengths of the terms $\mathcal{A}_{i;jk}$ and $\mathcal{V}_{i;jk}$ are straightforward from their analytical expressions. We express them here for $\mathcal{A}_{i;jk}$:

**Proposition S.3:**

$$\frac{\partial \mathcal{A}_{i;jk}}{\partial r_{ij}} = 2r_i\left(\lambda_{i;j}\theta_{ij;k} + h_{i;j}\frac{\partial \theta_{ij;k}}{\partial r_{ij}} + h_{i;k}\frac{\partial \theta_{ik;j}}{\partial r_{ij}} - r_i(\frac{\partial \theta_{ij;k}}{\partial r_{ij}} + \frac{\partial \theta_{ik;j}}{\partial r_{ij}} + \frac{\partial \psi_i}{\partial r_{ij}})\right), \quad \text{(S.13)}$$

$$\frac{\partial \mathcal{A}_{i;jk}}{\partial r_{ik}} = 2r_i\left(h_{i;j}\frac{\partial \theta_{ij;k}}{\partial r_{ik}} + \lambda_{i;k}\theta_{ik;j} + h_{i;k}\frac{\partial \theta_{ik;j}}{\partial r_{ik}} - r_i(\frac{\partial \theta_{ij;k}}{\partial r_{ik}} + \frac{\partial \theta_{ik;j}}{\partial r_{ik}} + \frac{\partial \psi_i}{\partial r_{ik}})\right), \quad \text{(S.14)}$$

$$\frac{\partial \mathcal{A}_{i;jk}}{\partial r_{jk}} = 2r_i\left(h_{i;j}\frac{\partial \theta_{ij;k}}{\partial r_{jk}} + h_{i;k}\frac{\partial \theta_{ik;j}}{\partial r_{jk}} - r_i(\frac{\partial \theta_{ij;k}}{\partial r_{jk}} + \frac{\partial \theta_{ik;j}}{\partial r_{jk}} + \frac{\partial \psi_i}{\partial r_{jk}})\right), \quad \text{(S.15)}$$

with similar expressions for the derivatives of $\mathcal{V}_{i;jk}$. Note that those derivatives require that that the corresponding derivatives of the dihedral angles they include are known. Computations of those derivatives are provided in section D below.

## Intersection of four balls

The formula for the weighted area and volume of the contribution of a ball to the union of balls removes all contributions of the intersections of four balls, with the exception of a term $\text{vol}\, F_{i;jkl}$, which we describe here. Let $B_i, B_j, B_k, B_l$ have a non-empty common intersection. Their centers define a tetrahedron, $T$, with faces $T_i, T_j, T_k, T_l$, defined such that $z_a \notin T_a$ for all $a = i, j, k, l$. We denote the dihedral angle of $T$ between the two faces that share the edge $z_i z_j$ by $\phi_{ij;kl}$. Let $F_{i;jkl}$ be the region delimited by the tetrahedron $T$ and the three Voronoi planes that separate $B_i$ from $B_j, B_k, B_l$.

**Proposition S.4:** The volume of $F_{i;jkl}$ is

$$\begin{aligned}\operatorname{vol} F_{i;jkl} = &\frac{1}{6}(r_i - h_{i;j})r_{ij}^2 \frac{2\cos\theta_{ij;k}\cos\theta_{ij;l} - (\cos^2\theta_{ij;k} + \cos^2\theta_{ij;l})\cos\phi_{ij;kl}}{\sin\phi_{ij;kl}} \\ &+\frac{1}{6}(r_i - h_{i;k})r_{ik}^2 \frac{2\cos\theta_{ik;j}\cos\theta_{ik;l} - (\cos^2\theta_{ik;j} + \cos^2\theta_{ik;l})\cos\phi_{ik;jl}}{\sin\phi_{ik;jl}} \\ &+\frac{1}{6}(r_i - h_{i;l})r_{il}^2 \frac{2\cos\theta_{il;j}\cos\theta_{il;k} - (\cos^2\theta_{il;j} + \cos^2\theta_{il;k})\cos\phi_{il;jk}}{\sin\phi_{il;jl}},\end{aligned} \tag{S.16}$$

in which the angles $\theta$ have been defined in the previous section. See Mach and Koehl, 2011[4] for the proof. Formulas for the derivatives of the term $\operatorname{vol} F_{i;jkl}$ with respect to the lengths of the six edges of $T$ are straightforward from this analytical expression.

## Derivatives of $\gamma_i$, $\gamma_{ij}$, and $\gamma_{ijk}$

The angular coefficient, $\gamma_i$, of a vertex $z_i$ is computed over all tetrahedra of $K$ that contain $i$. If $z_i$ is such that it belongs to at least one tetrahedron of $K$ that also contains $z_a$ and $z_b$, then

$$\frac{\partial \gamma_i}{\partial r_{ab}} = -\frac{1}{4\pi} \sum_{j|s_{ijab} \in K} \left( \frac{\partial \phi_{ij;ab}}{\partial r_{ab}} + \frac{\partial \phi_{ia;jb}}{\partial r_{ab}} + \frac{\partial \phi_{ib;ja}}{\partial r_{ab}} \right). \tag{S.17}$$

In all other cases, $\frac{\partial \gamma_i}{\partial r_{ab}} = 0$. Similarly,

$$\frac{\partial \gamma_{ij}}{\partial r_{ab}} = -\frac{1}{2\pi} \frac{\partial \phi_{ij;ab}}{\partial r_{ab}} \tag{S.18}$$

if $\tau_{ijab} \in K$, and $0$ otherwise. The derivatives of the dihedral angles of a tetrahedron with respect to its edge lengths are given in section D below. The derivatives of $\gamma_{ijk}$ are piecewise zero because the $\gamma_{ijk}$ are piecewise constant. Their values change at non-generic states, where their derivatives are not defined.[5,6]

# B. Integrated Mean Curvature, and Derivative

Recall that the mean curvature is the sum of two terms: the contribution of the spherical patches and of the accessible circular arcs at the intersections of pairs of spheres. Along these arcs, the mean curvature is partitioned equally between the two spheres involved. The contribution of a ball $i$ to the total mean curvature of a union of balls is then

$$M_i \;=\; \frac{A_i}{r_i} - \frac{\pi}{2} \sum_{j|s_{ij} \in K} \sigma_{ij} \alpha_{ij} r_{i;j}, \tag{S.19}$$

in which $A_i$ is the contribution of $B_i$ to the total surface area of the union of balls. Expressions for $A_i$ and its derivatives have been given above. Here we are concerned with the three additional terms: $r_{i;j}$, $\alpha_{ij}$, and $\sigma_{ij}$. Recall that $r_{ij}$ is the radius of the circle $S_{ij}$ at which $S_i$ and $S_j$ intersect; see Figure S.1. Its value and derivative with respect to the distance between the centers of $S_i$ and $S_j$ are provided in Equations (S.3) and (S.6).

## Derivative of $\alpha_{ij}$

The angle between the unit normals of $S_i$ and $S_j$ at any point $P$ on the circle $S_{ij}$ is denoted $\alpha_{ij}$. Using the law of cosine in the triangle $\Delta z_i z_j P$, we get

$$\alpha_{ij} = \arccos \frac{r_i^2 + r_j^2 - r_{ij}^2}{2 r_i r_j}. \tag{S.20}$$

A straightforward differentiation of Equation (S.20) gives the derivatives of $\alpha_{ij}$ with respect to the distance, $r_{ij}$, between the centers $z_i$ and $z_j$ of $S_i$ and $S_j$:

$$\frac{\partial \alpha_{ij}}{\partial r_{ij}} = \frac{2 r_{ij}}{\sqrt{4 r_i^2 r_j^2 - (r_i^2 + r_j^2 - r_{ij}^2)^2}}. \tag{S.21}$$

## Derivative of $\sigma_{ij}$

Let $S_i$ and $S_j$ be two intersecting spheres, and let $S_{ij}$ at which they intersect. $\sigma_{ij}$ is the fraction of the length of $S_{ij}$ that is accessible, i.e. not covered by any other spheres. In references,[5,7] Edelsbrunner and co-workers developed extrinsic formulas for $\sigma_{ij}$ and its derivatives, which are based on the Cartesian coordinates of the centers of the spheres that intersect $S_{ij}$. Here we revisit this problems and derive intrinsic formulas based on the distances between the sphere centers. We first establish

**Proposition S.5:** The fraction of $S_{ij}$ that is not covered is given by

$$\sigma_{ij} = 1 - 2 \sum_{k|s_{ijk} \in K} \gamma_{ijk} \frac{\theta_{ij;k}}{2\pi} - \sum_{kl|s_{ijkl} \in K} \frac{\phi_{ij;kl}}{2\pi}, \qquad \text{(S.22)}$$

in which the angles $\theta_{ij;k}$ and $\phi_{ij;kl}$ are the dihedral angles at the edge $z_i z_j$ in the tetrahedra $T_3 = z_i z_j z_k P_{ijk}$ and $T_4 = z_i z_j z_k z_l$ (see section above for details), and $\gamma_{ijk}$ is defined as

$$\gamma_{ijk} = 1 - \sum_{l|\tau_{ijkl} \in K} \frac{1}{2}. \qquad \text{(S.23)}$$

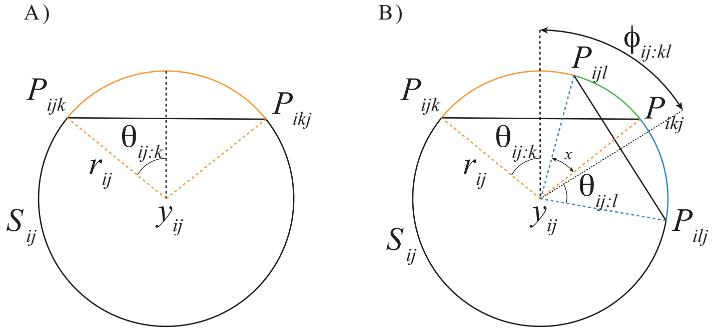


Figure S.3: The circle of intersection of two spheres. We consider two spheres, $S_i$ and $S_j$, with non-empty intersection. The Voronoi plane between their centers cuts the two spheres in a circle with center $y_{ij}$ and radius $r_{ij}$. In **A**, this circle is partially covered by a third sphere, $S_k$, that intersects the circle at two points $P_{ijk}$ and $P_{ijkl}$. In **B**, the circle is partially covered by two sphere $S_k$ and $S_l$. We assume that the corresponding caps between $P_{ijk}$ and $P_{ikj}$ for sphere $S_k$, and between $P_{ijl}$ and $P_{ilj}$ for sphere $S_l$, are not nested. This is always the case if the spheres arise from triangles in the dual complex.

The proof follows from the inclusion-exclusion principle. We illustrate it here for two, three, and four spheres. In the simplest case of two intersecting spheres, the entire length of the circle is accessible, and $\sigma_{ij} = 1$. When three spheres, $S_i, S_j, S_k$, intersect in an accessible point, then they form a triangle in the dual complex, $K$, and $S_{ij}$ is partially covered by $S_k$; see Figure S.3A. The arc $S_{ij}$ that is covered connects the points $P_{ijk}$ and $P_{ikj}$, and its length is $r_{ij} \cdot 2\theta_{ij;k}$. In this case,

$$\sigma_{ij} = \frac{2\pi r_{ij} - 2r_{ij}\theta_{ij;k}}{2\pi r_{i;j}} = 1 - \frac{\theta_{ij;k}}{\pi}. \tag{S.24}$$

When four spheres, $S_i, S_j, S_k, S_l$ intersect in such a way that they form a tetrahedron in the dual complex, $K$, then $S_{ij}$ is partially covered by both $S_k$ and $S_l$; see Figure S.3B. The length of $S_{ij}$ that is covered is the sum of arc $P_{ijk}P_{ikj}$, whose length is $r_{ij} \cdot 2\theta_{ij;k}$, and the arc $P_{ijl}P_{ilj}$, whose length is $r_{ij} \cdot 2\theta_{ij;l}$, minus the sub arc common to those two arcs, whose length is $r_{ij} \cdot x$. Notice that $x = \theta_{ij;k} + \theta_{ij;l} - \phi_{ij;kl}$. Therefore

$$\begin{aligned}\sigma_{ij} &= \frac{2\pi r_{ij} - 2r_{ij}\theta_{ij;k} - 2r_{ij}\theta_{ij;l} + r_{ij}x}{2\pi r_{ij}} \\ &= \frac{2\pi r_{ij} - r_{ij}\theta_{ij;k} - r_{ij}\theta_{ij;l} - r_{ij}\phi_{ij;kl}}{2\pi r_{ij}} \\ &= 1 - \frac{\theta_{ij;k}}{2\pi} - \frac{\theta_{ij;l}}{2\pi} - \frac{\phi_{ij;kl}}{2\pi}.\end{aligned} \tag{S.25}$$

The extensions of the three cases to include all simplices of the dual complex that contain the edge $z_i z_j$ leads to Equation (S.22). Formulas for the derivatives of $\sigma_{ij}$ with respect to edge lengths are then straightforward:

$$\frac{\partial \sigma_{ij}}{\partial r_{ab}} = 1 - \frac{1}{\pi} \sum_{k|\tau_{ijk} \in K} \gamma_{ijk} \frac{\partial \theta_{ij;k}}{\partial r_{ab}} - \frac{1}{2\pi} \sum_{kl|\tau_{ijkl} \in K} \frac{\partial \phi_{ij;kl}}{\partial r_{ab}}, \tag{S.26}$$

in which the derivatives of the dihedral angles of a tetrahedron as a function of edge lengths are provided in section D below.

# C. Integrated Gaussian Curvature, and Derivative

Recall that the Gaussian curvature is the sum of three terms that account for the spherical patches, the circular arcs between spheres, and corners on the boundary of the union of balls:

$$G_i \;=\; \frac{A_i}{r_i^2} - \frac{\pi}{2} \sum_{j|s_{ij}\in K} \sigma_{ij}\lambda_{ij} + \frac{1}{3} \sum_{j,k|s_{ijk}\in K} \gamma_{ijk}\sigma_{i;jk}. \tag{S.27}$$

All variables have been defined above, with the exception of $\lambda_{ij}$ and $\sigma_{i;jk}$, which we describe below.

## Derivative of $\lambda_{ij}$

$\lambda_{ij}$ is the distance between the projections of the unit normals of $S_i$ and $S_j$ at a point, $P$, on the circle of intersection, $S_{ij}$; see Figure S.1. It can be computed from the signed distances, $\zeta_i$ and $\zeta_j$, of the centers, $z_i$ and $z_j$, from the Voronoi plane that passes through $S_{ij}$; see Equation (S.1):

$$\lambda_{ij} = \frac{\zeta_i}{r_i} + \frac{\zeta_j}{r_j} = \frac{r_{ij}}{2}\left(\frac{1}{r_i} + \frac{1}{r_j}\right) + \frac{r_i^2 - r_j^2}{2r_{ij}}\left(\frac{1}{r_i} - \frac{1}{r_j}\right). \tag{S.28}$$

The derivative of $\lambda_{ij}$ with respect to $r_{ij}$, the distance between $z_i$ and $z_j$, is therefore

$$\frac{\partial\lambda_{ij}}{\partial r_{ij}} = \frac{1}{2}\left(\frac{1}{r_i} + \frac{1}{r_j}\right) - \frac{r_i^2 - r_j^2}{2r_{ij}^2}\left(\frac{1}{r_i} - \frac{1}{r_j}\right). \tag{S.29}$$

## Derivative of $\sigma_{i;jk}$

$\sigma_{i;jk}$ is the corner term that is specific to the Gaussian curvature. Such a corner exists for each accessible triangle of the dual complex, and there are two corners for each triangle that is accessible from both sides. Let us consider one such triangle, with vertices $z_i, z_j, z_k$, which are the centers of the spheres $S_i, S_j, S_k$. These three spheres intersect at two corners, $P_{ijk}$ and $P_{ikj}$ (see Figure S.2). As the spheres may have different radii, we need a scheme to divide the contribution of the corner to the Gaussian curvature among the three spheres, and to compute the derivatives

of the sphere-specific contributions. In agreement with the general approach used in this paper,

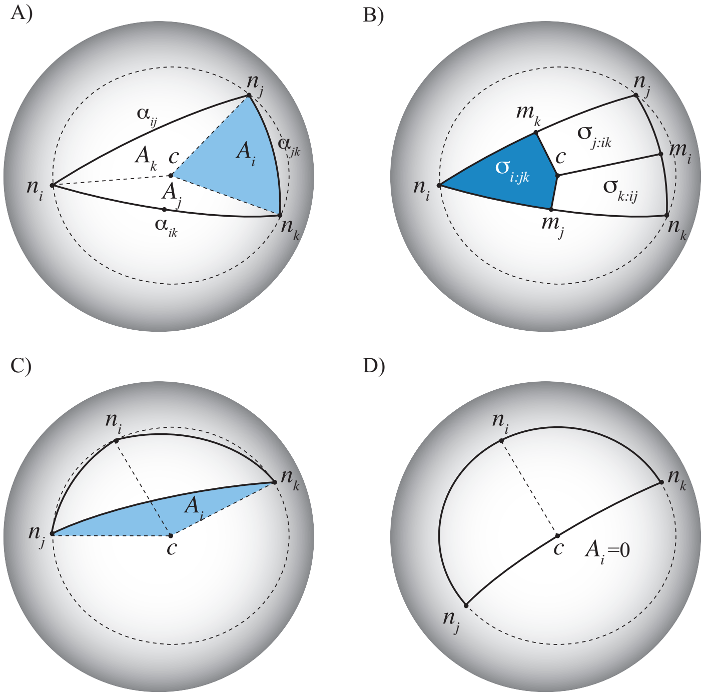


Figure S.4: A corner contributing to the Gaussian curvature. We consider three intersecting spheres, $S_i, S_j, S_k$, and one of the point, $P_{ijk}$, common to the three spheres. **A**) The outward unit normals, $n_i, n_j, n_k$, at this point form a spherical triangle on the unit sphere whose area is the contribution of $P_{ijk}$ to the Gaussian curvature. To divide this contribution, we use the center, $c$, of the unique circle that passes through $n_i, n_j, n_k$. **B**) The spherical triangle is divided into three quadrangles by connecting $c$ to the midpoints of the three edges. The area $\sigma_{i;jk}$ is the contribution to the Gaussian curvature we associate with $S_i$. **C**) Note that $c$ may be outside of the spherical triangle $n_i n_j n_k$. In the example shown, the oriented area, $A_i$, of $n_j c n_k$ is negative. **D**) In the limiting case, $c$ lies on an edge of the spherical triangle, here $n_j n_k$, which implies $A_i = 0$. This special case leads to a singularity that needs to be handled explicitly (see text for details).

all these derivatives will be expressed as functions of the inter-vertex distances, which in this case are $r_{ij}, r_{jk}, r_{ik}$. These formulas have been derived in Akopyan and Edelsbrunner, 2020.[8] Fix $P_{ijk}$ and let $n_i, n_j, n_k$ be the unit outward normals of the three spheres at $P_{ijk}$. The total contribution of this corner to the Gaussian curvature is equal to the area $\sigma_{ijk}$ of the spherical triangle with vertices $n_i, n_j, n_k$ on the unit sphere; see Figure S.4. The geodesic lengths of the sides are $\alpha_{ij}, \alpha_{jk}, \alpha_{ik}$, as defined in Figure S.1. We begin with the following

**Proposition S.6:** Let $T$ be a spherical triangle with side lengths $\alpha, \beta, \gamma$, and set $a = \cos^2(\alpha/2)$, $b = \cos^2(\beta/2)$, $c = \cos^2(\gamma_{ik}/2)$. The area of this triangle is

$$S(a,b,c) = 2 \arcsin \sqrt{\frac{4abc - (a+b+c-1)^2}{4abc}}. \tag{S.30}$$

In addition, if $R(a,b,c)$ is the radius of the circumcircle and $r(a,b,c) = \cos^2(R(a,b,c)/2)$, then

$$r(a,b,c) = \frac{1}{2} + \frac{1}{2}\sqrt{\frac{4abc - (a+b+c-1)^2}{4(1-a)(1-b)(1-c) + 4abc - (a+b+c-1)^2}}. \tag{S.31}$$

Equation (S.30) was established in Akopyan and Edelsbrunner 2020[8] with a somewhat cumbersome proof. We provide here a simpler proof. Starting with a formula for the cosine of half of the area that was originally established by Euler:[9,10]

$$\cos\frac{S(a,b,c)}{2} = \frac{1 + \cos\alpha + \cos\beta + \cos\gamma}{4\cos\frac{\alpha}{2}\cos\frac{\beta}{2}\cos\frac{\gamma}{2}}. \tag{S.32}$$

Using $\cos x = 2\cos^2\frac{x}{2} - 1$ and the definitions of $a, b, c$, we get,

$$\cos\frac{S(a,b,c)}{2} = \frac{a+b+c-1}{2\sqrt{abc}}. \tag{S.33}$$

Therefore,

$$\sin\frac{S(a,b,c)}{2} = \sqrt{1 - \cos^2\frac{S(a,b,c)}{2}} = \sqrt{\frac{4abc - (a+b+c-1)^2}{4abc}}, \tag{S.34}$$

which implies (S.30). Equation (S.31) is derived from the relationship between the radius of the circumcircle and the area of the triangle:[11]

$$\tan R(a,b,c) = \frac{\tan\frac{\alpha}{2}\tan\frac{\beta}{2}\tan\frac{\gamma}{2}}{S(a,b,c)}. \tag{S.35}$$

This completes the proof. As a consequence of Proposition S.6, $\sigma_{ijk} = S(a,b,c)$, is the total

contribution of the corner $P_{ijk}$ to the Gaussian curvature. Since $S_i, S_j, S_k$ have different weights, we break down this contribution to individual contributions of the spheres: $\sigma_{ijk} = \sigma_{i;jk} + \sigma + j : ki + \sigma_{k;ij}$, in which $\sigma_{i;jk} = \omega_i \sigma_{ijk}$, etc. The division is based on the position of the spherical circumcenter, $c$, of $n_i, n_j, n_k$; see S.4A for details. Note that the coefficients $\omega_i, \omega_j, \omega_k$ can be seen as spherical barycentric coordinates of $c$ with respect to the spherical triangle $n_i n_j n_k$. To compute these coordinates, we split $n_i n_j n_k$ in two different ways. First, the triangle is split into three triangles, $cn_j n_k$, $n_i cn_k$, $n_i cn_k$, with surface areas $A_i, A_j, A_k$. Let $R(a,b,c)$ is the spherical radius of the circumcircle of $n_i, n_j, n_k$, and with $r(a,b,c) = \cos^2(R(a,b,c)/2)$ we get

$$\begin{aligned} A_i &= S(r,r,c), \\ A_j &= S(r,b,r), \\ A_k &= S(a,r,r). \end{aligned} \tag{S.36}$$

Second, we let $m_i, m_j, m_k$ be the midpoints of $z_j z_k, z_i z_k, z_i z_j$, and we subdivide $n_i n_j n_k$ into three quadrangles, $n_i m_k c m_j$, $n_j m_k c m_i$, $n_k m_j c m_i$, with areas $\sigma_{i;jk}$, $\sigma_{j;ik}$, and $\sigma_{k;ij}$, respectively. To establish the correspondence between the areas $A$ and the areas $\sigma$, we need to take into account the possibility that $c$ falls outside of the triangle $n_i n_j n_k$. In the case illustrated in Figure S.4C, the corresponding spherical barycentric coordinate $\omega_i$ would be negative. This occurs when $n_i$ and $c$ lie on opposite side of the side $n_j n_k$. The boundary case, when $c$ lies on $n_j n_k$ (Figure S.4D) occurs when $\sin^2(\alpha_{ij}/2) + \sin^2(\alpha_{ik}/2) = \sin^2(\alpha_{jk}/2)$ or, equivalently, when $a + c = 1 + b$ (see Akopyan and Edelsbrunner, 2020[8] for details). When $a + c \leq 1 + b$, $n_i$ and $c$ lie on the same side of $n_j n_k$. We define

$$\operatorname{sgn}(i, jk) = \begin{cases} +1 & \text{if } a + c \leq 1 + b \\ -1 & \text{otherwise.} \end{cases} \tag{S.37}$$

Using this sign function, we get:

$$\sigma_{i;jk} = \frac{1}{2}[\mathrm{sgn}(k, ij)A_k + \mathrm{sgn}(j, ik)A_j], \tag{S.38}$$

$$\sigma_{j;ik} = \frac{1}{2}[\mathrm{sgn}(i, jk)A_i + \mathrm{sgn}(k, ij)A_k], \tag{S.39}$$

$$\sigma_{k;ij} = \frac{1}{2}[\mathrm{sgn}(i, jk)A_i + \mathrm{sgn}(j, ik)A_j]. \tag{S.40}$$

The radius $R(a, b, c)$ of the circumcircle, or equivalently, the cosine squared of its half, $r(a, b, c)$, are computed based on Equation (S.31). Finally, the derivatives of Equations (S.38) to (S.40) are derived by simple chain rules using the analytical expressions for $S(a, b, c)$ and $r(a, b, c)$, as well as the derivatives of the angles $\alpha$ as a function of edge lengths, provided in Equation (S.21).

## A singularity when computing the derivatives

To compute the derivatives of the different surface areas, $\sigma_{i;jk}, \sigma_{j;ik}, \sigma_{k;ij}$, we need the derivatives of the areas $A_i, A_j, A_k$. These terms are all computed as surface areas of spherical triangles, given by Proposition S.6. We recall Equation 21 in Akopyan and Edelsbrunner, 2020:[8]

$$\frac{\partial S(a, b, c)}{\partial a} = \frac{-a + b + c - 1}{a\sqrt{4abc - (a + b + c - 1)^2}}. \tag{S.41}$$

Using Proposition S.6, we rewrite it as

$$\frac{\partial S(a, b, c)}{\partial a} = \frac{-a + b + c - 1}{a\sqrt{4abc}\sin\frac{S(a,b,c)}{2}}. \tag{S.42}$$

Difficulties arise when the center, $c$, of the circle that passes through $n_i, n_j, n_k$ lies on one of the edges of the spherical triangle; see Figure S.4D. Let us assume, for example, that $c$ lies on the edge $n_j n_k$. Then $A_i = 0$. According to Equation (S.42), however,

$$\frac{\partial A_i}{\partial c} = \frac{-c + 2r - 1}{a\sqrt{4cr^2}\sin\frac{A_i}{2}}. \tag{S.43}$$

As $A_i$ is zero, we cannot use this formula. Instead, none of the terms $S(a, b, c)$, $A_j$, and $A_k$ can be zero in this case. Therefore, we can compute their derivatives, and by summing Equations (S.38) to (S.40), we get

$$S(a, b, c) = \operatorname{sgn}(k, ij)A_k + \operatorname{sgn}(i, jk)A_i + \operatorname{sgn}(j, ik)A_j. \tag{S.44}$$

Using this equation, we derive

$$\operatorname{sgn}(i, jk)\frac{\partial A_i}{\partial c} = \frac{\partial S(a, b, c)}{\partial c} - \operatorname{sgn}(j, ik)\frac{\partial A_j}{\partial c} - \operatorname{sgn}(k, ij)\frac{\partial A_k}{\partial c}, \tag{S.45}$$

with similar expressions for the derivatives with respect to $a$ and $b$.

# D. The Geometry of a Tetrahedron

Let us consider the tetrahedron, $T$, with vertices $P_1, P_2, P_3, P_4$. The four faces of $T$ are $T_1 = P_2P_3P_4$, $T_2 = P_1P_3P_4$, $T_3 = P_1P_2P_4$, and $T_4 = P_1P_2P_3$, and we write $s_1, s_2, s_3, s_4$ for their areas, respectively. We denote the dihedral angle between $T_i$ and $T_j$ as $\theta_{ij}$ and the length of the edge connecting $P_i$ and $P_j$ as $r_{ij}$.

## Volume and Surface area

The Cayley-Menger matrix, $M$, associated with the tetrahedron, $T$, is

$$M = \begin{pmatrix} 0 & r_{12}^2 & r_{13}^2 & r_{14}^2 & 1 \\ r_{12}^2 & 0 & r_{23}^2 & r_{24}^2 & 1 \\ r_{13}^2 & r_{23}^2 & 0 & r_{34}^2 & 1 \\ r_{14}^2 & r_{24}^2 & r_{34}^2 & 0 & 1 \\ 1 & 1 & 1 & 1 & 0 \end{pmatrix}. \tag{S.46}$$

We write $M_{ij}$ for the submatrix obtained by deleting the $i$-th row and the $j$-th column of $M$. The volume $V$ of $T$ and its surface areas can be expressed in terms of the determinants of these matrices:

$$V^2 = \frac{1}{288}\det(M), \tag{S.47}$$

$$s_i^2 = -\frac{1}{16}\det(M_{ii}), \tag{S.48}$$

in which

$$\det(M_{ij}) = 2r_{ij}^2(r_{ik}^2 + r_{il}^2 - r_{kl}^2) - (r_{ij}^2 + r_{ik}^2 - r_{jk}^2)(r_{ij}^2 + r_{il}^2 - r_{jl}^2). \tag{S.49}$$

## Dihedral angles

We use the law of cotangents of dihedral angles[4] to express the relationship between the above two determinants and the dihedral angles of the tetrahedron:

$$\cot\theta_{ij} = \frac{1}{24}\frac{(-1)^{i+j}\det(M_{ij})}{r_{ij}V}. \tag{S.50}$$

## Derivative of the volume of a tetrahedron

**Proposition D.1:** Let $T$ be a non-degenerate tetrahedron with volume $V$. The derivative of $V$ with respect to the length $r_{ab}$ of the edge $P_aP_b$ is

$$\frac{\partial V}{\partial r_{ab}} = \frac{1}{6}r_{ab}^2\cot\theta_{ab}. \tag{S.51}$$

See[4] for the proof.

## Derivative of the dihedral angles of a tetrahedron

Deriving Equation (S.50) with respect to the length, $r_{ab}$, of the edge connecting the vertices $P_a$ and $P_b$, we get

$$-(1+\cot^2\theta_{ij})\frac{\partial\theta_{ij}}{\partial r_{ab}} = -\delta_{ij;ab}\frac{\cot\theta_{ij}}{r_{ij}} + \frac{1}{24}\frac{(-1)^{i+j}}{r_{ij}V}\frac{\partial \det(M_{ij})}{\partial r_{ab}} - \frac{r_{ab}^2\cot\theta_{ij}\cot\theta_{ab}}{6V}, \quad \text{(S.52)}$$

in which $\delta_{ij;ab}$ is 1 if the pair $(i,j)$ is equal to the pair $(a,b)$ and equal to 0 otherwise. All terms in this equation are known, with the derivatives of $\det(M_{ij})$ being computed from Equation (S.49).

# E. Database of Virus Capsid Structures

Table S.1: List of viruses: PDB file and size

| PDB [a)] | size [b)] | PDB | size | PDB | size | PDB | size |
|---|---|---|---|---|---|---|---|
| 1al0 | 571260 | 1ihm | 677040 | 1vbd | 400500 | 2q25 | 454800 |
| 1al2 | 432180 | 1k4r | 545040 | 1w8x | 2286960 | 2q26 | 460140 |
| 1ar6 | 432540 | 1ld4 | 940800 | 1z7s | 405900 | 2qqp | 998040 |
| 1ar7 | 432720 | 1m1c | 618120 | 1 z7z | 529440 | 2tbv | 421380 |
| 1ar8 | 431040 | 1m4x | 16284240 | 2bbv | 469020 | 2vf1 | 497160 |
| 1ar9 | 432300 | 1nov | 447060 | 2bld | 5214540 | 2w0c | 1586820 |
| 1asj | 432480 | 1ohf | 1071300 | 2btv | 2943660 | 2z2q | 455580 |
| 1b35 | 413520 | 1ohg | 904200 | 2bvi | 5209380 | 2zah | 425700 |
| 1cd3 | 591060 | 1po1 | 401400 | 2dum | 5214540 | 3ddx | 579180 |
| 1ej6 | 2069220 | 1po2 | 401280 | 2frp | 877860 | 3e8k | 815400 |
| 1f8v | 523140 | 1qgc | 496740 | 2fs3 | 879840 | 3gzt | 1568580 |
| 1gw7 | 2050200 | 1rvf | 481140 | 2fsy | 877860 | 3gzu | 3239460 |
| 1gw8 | 2050200 | 1sid | 1020180 | 2ft1 | 886620 | 3j27 | 882540 |
| 1hb5 | 1636380 | 1sie | 1028460 | 2fte | 563220 | 3j3a | 2440800 |
| 1hb7 | 2050800 | 1sva | 958980 | 2gh8 | 764820 | 3lob | 464400 |
| 1hb9 | 2181840 | 1uf2 | 3487800 | 2plv | 429720 | 5iz7 | 793560 |
| 1hxs | 435360 | 1upn | 447780 | 2q23 | 454620 | 5j7v | 26251097 |

[a)] PDB[12] code for the structure recovered at `https://www.rcsb.org` (the biological assembly that contains the full capsid).

[b)] Total number of atoms in the full capsid.

# References


(1) Gibson, K. D.; Scheraga, H. A. Exact calculation of the volume and surface-area of fused hard-sphere molecules with unequal atomic radii. *Mol. Phys.* **1987**, *62*, 1247–1265.

(2) Gibson, K. D.; Scheraga, H. A. Surface area of the intersection of three spheres with unequal radii. A simplified formula. *Mol. Phys.* **1988**, *64*, 641–644.

(3) Edelsbrunner, H.; Fu, P. Measuring space filling diagrams and voids. *Technical Report, Univ. Illinois, Urbana, Illinois* **1994**, *UIUC-BI-MB-94-01*.

(4) Mach, P.; Koehl, P. Geometric measures of large biomolecules: surface, volume, and pockets. *J. Comput. Chem.* **2011**, *32*, 3023–3038.

(5) Bryant, R.; Edelsbrunner, H.; Koehl, P.; Levitt, M. The area derivative of a space-filling diagram. *Discrete Comput. Geom.* **2004**, *32*, 293–308.

(6) Edelsbrunner, H.; Koehl, P. The weighted-volume derivative of a space-filling diagram. *Proc. Natl. Acad. Sci. (USA)* **2003**, *100*, 2203–2208.

(7) Akopyan, A.; Edelsbrunner, H. The Weighted Mean Curvature Derivative of a Space-Filling Diagram. *Comput. Math. Biophys.* **2020**, *8*, 51–67.

(8) Akopyan, A.; Edelsbrunner, H. The Weighted Gaussian Curvature Derivative of a Space-Filling Diagram. *Comput. Math. Biophys.* **2020**, *8*, 74–88.

(9) Euler, L. Variae speculationes super area triangulorum sphaericorum, Nova Acta academiae scientarum imperialis Petropolitinae 10. *Opera Omni Series 1* **1797**, *XXIX*, 253–266.

(10) Papadopoulos, A. On the works of Euler and his followers on spherical geometry. *arXiv e-prints* **2014**, ArXiv:1409.4736.

(11) Chauvenet, W. *Treatise on plane and spherical trigonometry, 9th edition*; Lippincott Company: Philadelphia, PA, 1887.

(12) Berman, H. M.; Westbrook, J.; Feng, Z.; Gilliland, G.; Bhat, T. N.; Weissig, H.; Shindyalov, I.; Bourne, P. The Protein Data Bank. *Nucl. Acids. Res.* **2000**, *28*, 235–242.
